# Social waves in giant honeybees (*Apis dorsata*) elicit nest vibrations

**DOI:** 10.1007/s00114-013-1056-z

**Published:** 2013-05-31

**Authors:** Gerald Kastberger, Frank Weihmann, Thomas Hoetzl

**Affiliations:** Department of Zoology, University Graz, 8010 Graz, Austria

**Keywords:** Giant honeybee, *Apis dorsata*, Bee curtain, Shimmering, Defence waves, Comb vibrations, Elastic plate model, Mechanic pendulum model, Frequency spectrum, Colony-intrinsic communication

## Abstract

Giant honeybees (*Apis dorsata*) nest in the open and have developed a wide array of strategies for colony defence, including the Mexican wave-like shimmering behaviour. In this collective response, the colony members perform upward flipping of their abdomens in coordinated cascades across the nest surface. The time–space properties of these emergent waves are response patterns which have become of adaptive significance for repelling enemies in the visual domain. We report for the first time that the mechanical impulse patterns provoked by these social waves and measured by laser Doppler vibrometry generate vibrations at the central comb of the nest at the basic (=‘natural’) frequency of 2.156 ± 0.042 Hz which is more than double the average repetition rate of the driving shimmering waves. Analysis of the Fourier spectra of the comb vibrations under quiescence and arousal conditions provoked by mass flight activity and shimmering waves gives rise to the proposal of two possible models for the compound physical system of the bee nest: According to the elastic oscillatory plate model, the comb vibrations deliver supra-threshold cues preferentially to those colony members positioned close to the comb. The mechanical pendulum model predicts that the comb vibrations are sensed by the members of the bee curtain in general, enabling mechanoreceptive signalling across the nest, also through the comb itself. The findings show that weak and stochastic forces, such as general quiescence or diffuse mass flight activity, cause a harmonic frequency spectrum of the comb, driving the comb as an elastic plate. However, shimmering waves provide sufficiently strong forces to move the nest as a mechanical pendulum. This vibratory behaviour may support the colony-intrinsic information hypothesis herein that the mechanical vibrations of the comb provoked by shimmering do have the potential to facilitate immediate communication of the momentary defensive state of the honeybee nest to the majority of its members.

**Electronic supplementary material:**

The online version of this article (doi:10.1007/s00114-013-1056-z) contains supplementary material, which is available to authorized users.

## Introduction

Giant honeybees (*Apis dorsata*) are one of the most ancient honeybee species (Ruttner 1988; Oldroyd and Wongsiri 2006; Kastberger et al. 2011a). They nest in the open (Seeley et al. 1982) and are, therefore, especially exposed to predators, such as mammals (Kastberger 1999), birds (Seeley et al. 1982; Kastberger and Sharma 2000) and wasps (Seeley et al. 1982; Kastberger et al. 2008, 2010). Their most prominent defensive behaviour against such threats is shimmering, which generates repetitive social waves with anti-predatory impact (Kastberger et al. 2008, 2010, 2012, 2013; Weihmann et al. 2012). In shimmering, bees at the nest surface, predominantly younger cohorts (Lerchbacher et al., submitted) in the quiescent regions peripheral to the mouth zone (Kastberger et al. 2011b), show simultaneous and cascaded actions (Schmelzer and Kastberger 2009) whereby their abdomens are flipped upwards at an angle between 20° and 120° (Kastberger et al. 2011a, b). These individual mechanical actions of surface bees emerge to a visual display of waves which have the potential to repel or at least scare external predators such as wasps (Kastberger et al. 2008) or vertebrates (Kastberger et al. 2013).

The mechanical expression of shimmering, represented in particular by its motion components in the *z*-direction (i.e. towards and away from the comb; Kastberger et al. 2011b) is still unclear in its ultimate goals. However, two mechanistic viewpoints can here be proposed:

- First, mechanical cues provoked by shimmering may be important for the rapidness of the wave propagation across the nest. Alternative strategies eventually processed by visual cues among adjacent neighbours, utilising stigmergic (Grasse 1959; Kastberger et al. 2013) principles, such as bucket-bridging (Kastberger et al. 2012) or eavesdropping (Peake 2005; Jones et al. 2011), would be much slower by one or two orders of magnitude than those which are actually observed in shimmering (Kastberger et al. 2012, 2013). The saltatoric principle of wave propagation (Kastberger et al. 2013), which may speed up the wave from a basically bucket-bridging process by a factor of 3, is supposed to be associated to the visual input of threatening cues and to the mechanical effects of the shimmering waves in the bee curtain.
- Second, shimmering could benefit the entirety of the colony by allowing colony-intrinsic propagation of information across the nest via its mechanical expression of wave components (Kastberger et al. 2011b). This proposition is summarized by the colony-intrinsic information hypothesis which predicts that the shimmering waves may also disseminate information about the momentary defence state of the colony to those nest members which do not participate in this collective action. Shimmering-passive cohorts usually make up more than 90 % of the colony and comprise curtain bees in the surface and subsurface layers on both sides of the nest.

In accordance with this proposition of a potential colony-intrinsic spread of information across the nest, the mechanical components of the shimmering process should affect the centrally positioned comb of the nest. The comb is the only architectural structure of an *A. dorsata* nest with the capacity to rapidly bridge information from the threatened to the non-threatened nest side. Therefore, we examined whether shimmering does produce vibrations at the comb. To qualify as signals which could potentially be utilised for intra-colonial communication, mechanical vibrations of the comb should exceed at least the perception threshold of Western honeybees (Sandeman et al. 1996) which can be estimated at dislocation amplitudes of >9 μm in the low-frequency range (<10 Hz).

## Material and methods

### Experimental site

**Fig. 1:**
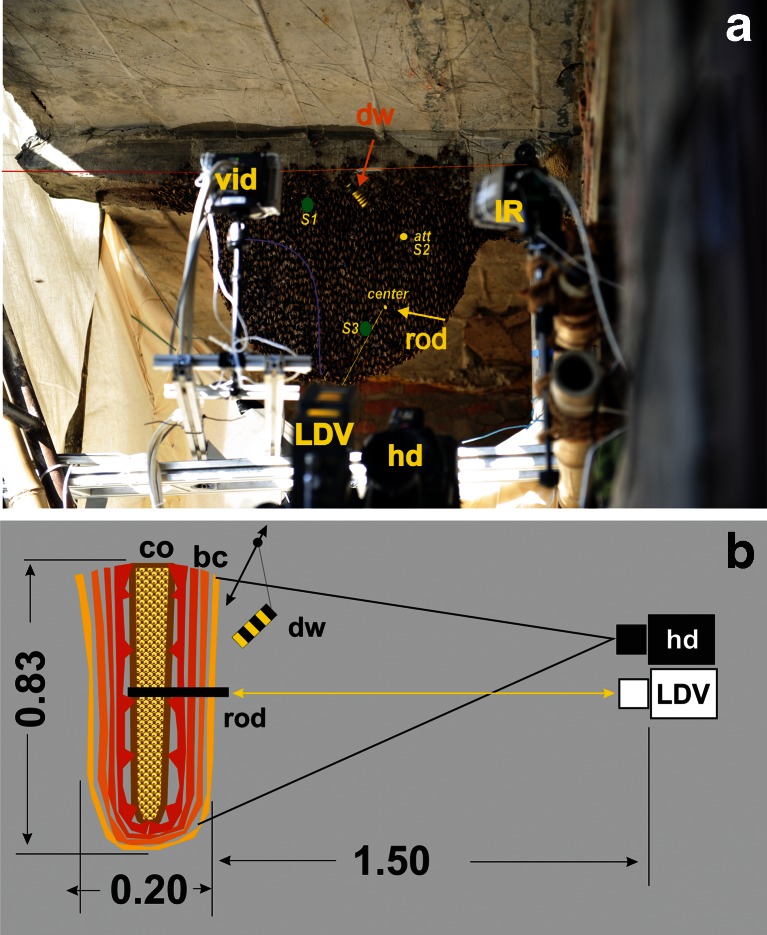
Measurement set-up of the experimental *A. dorsata* nest. **a** *hd* HD video camera, *vid* black and white video camera, *IR* infrared camera, *LDV* laser Doppler vibrometer, with the *yellow straight line* as the ray of the laser; the dummy wasp (*dw*, see *orange arrow*) was moved under computer control from the left to the right side of the nest, the *orange line* at the upper rim of the nest gives the wire of the cable car device by which the dummy had been moved. The mouth zone was positioned to the left of the *violet curve*. **b** Schematics of the set-up from the side view, with a perpendicular cross-section through the nest; distances in metres; a wooden rod (diameter, 8 mm; length, 10 cm) was stuck through the bee curtain (*bc*) into the comb (*co*) at position *centre* or alternatively at positions S1–3 with S2 = *att*, and a white plate was mounted on the front plane of the rod to reflect the laser beam

The experimental nests of giant honeybees (*A. dorsata*) were located at a hotel site in Sauraha, Chitwan, Nepal (at the border of the Chitwan National Park). Preliminary experiments had been performed in February 2009, but in November 2010, a single nest had been selected for a much broader in-depth investigation. This nest was attached to blocks of concrete making up a balcony of the hotel. The nest had a hemicyclic form, measured 83 × 60 cm (width × height), was approximately 2 weeks old and could have had a weight of 30 kg (see Seeley et al. 1982 and the ‘Results’ section). In the first days of observation, the comb was covered only by a single layer of honeybees, but a few days later, the colony had achieved a multilayer cover due to the progressive hatching of young bees (Fig. 1).

### Video recording

The experimental nest was filmed with a high-definition (HD) video camera (Panasonic HVX 200) from a distance of 1.5 m, whereby the camera angle covered the whole nest (Fig. 1). This distance provided an undistorted view and was also distant enough to keep the colony undisturbed, as the bees accepted the camera as just a landmark. The recordings were made at 50 frames per second (FPS) with a resolution of 1,280 × 720 pixels.

### Laser vibrometry

A wooden rod (8 mm thick and 10 cm long with a weight of 1.28 g) was stuck into the comb, with one end protruding slightly out the surface of the bee curtain. A piece of white paper was glued to its plane end to serve as a reflector for the laser beam of the laser Doppler vibrometer (abbreviated further on as ‘LDV’; Polytec PDV 100; Fig. 1). In this way, the directional *z*-component of the movement of the comb (towards and away from the nest surface) was detected by the velocity values assessed by the Doppler phase shift between the emission and reflection of the laser signal. These data were assessed with the wooden rod positioned at selected points:

For the spectrum analysis, we used the positions S1–2 (14 November 2010) and included mass flight, pure quiescence and presentation of the dummy wasp. For further evaluations of the correlation between shimmering and comb vibrations, we used the position *att*, 10 cm below the attachment zone (experiments: 9 November 2010), and the position *centre*, central between the right-hand border of the mouth zone and the right-hand rim of the nest (experiments: 10 November 2010), at least 30 cm below the attachment zone (Fig. 1).

The LDV had a resolution of <0.05 μm/s. The raw data were produced by the LDV in sample intervals of 20 μs (resulting in a sampling frequency of *f*_s_ = 1 / (20 × 10^−6^) [Hz]), processed by MATLAB through a low-pass Butterworth IIR filter with a cut-off frequency of *f*_LP_ = 250 Hz (with *f*_LP_ = 0.01 × *f*_s_ / 2) and displayed in data streams, frequency spectrograms (abscissa: experimental time; ordinate: frequency components; *z*-axis: power spectral density [PSD]) and single-sided Fourier spectra (abscissa: frequency components; ordinate: LDV signal [in millimetres per second]). The raw Butterworth filtered LDV data streams were offset-corrected and integrated for the calculation of dislocation values scaled in millimetres.

### Dummy wasp stimulation

**Fig. 2:**
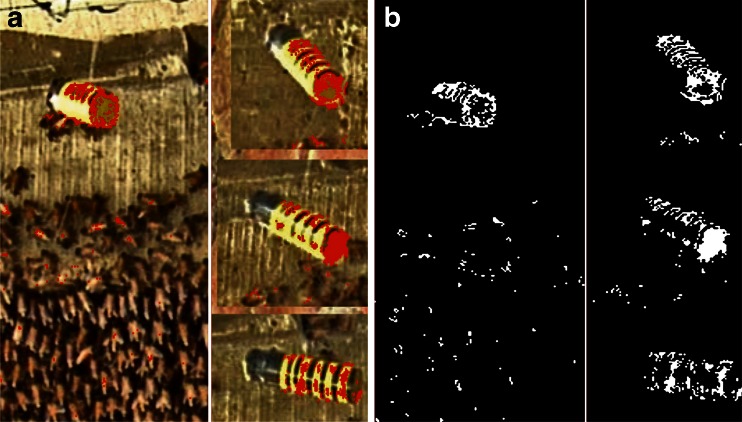
Automated detection of the dummy wasp position. Four scenes under various lighting conditions and angles taken from real video images of the dummy wasp. The colour of the dummy was partially matched in the video film (marked as *red areas* in **a**) and segmented (marked as *white areas* in **b**); for comparison, see HD images of the dummy above the attachment zone of the nest in Figs. 1 and 3

We stimulated the experimental colony by presenting a dummy wasp which was a cuboid Styrofoam block measuring 8 × 2 × 2 cm covered with yellow and black stripes (Fig. 1; Kastberger et al. 2011a, b, 2012, 2013). It was suspended beneath a horizontal wire fixed above the nest attached by a flexible thread, allowing the dummy to swing. It was moved by a miniature computer-driven cable car device at a constant velocity (0.1–0.5 m/s) along the horizontal wire, 20 cm in front of the nest near its upper attachment zone. This stimulation method was chosen to mimic a free-flying wasp scanning in front of the nest and has regularly provoked shimmering (Kastberger et al. 2011a, b). The dummy wasp (dw) was presented to the colony by drawing it from the parking site at the upper left side of the nest to the right side and back again (Fig. 1). Automated pinpointing of the dummy’s location was achieved by assessment of its motion (see the next section) using predefined templates. This allowed the identification of the dummy regardless of the viewing angle and the associated light conditions (Fig. 2) and the assessment of its gravity position (dummy: *x*_dw_, *y*_dw_) during the whole presentation cycle.

### Experimental sessions and assessment of shimmering motions

**Fig. 3:**
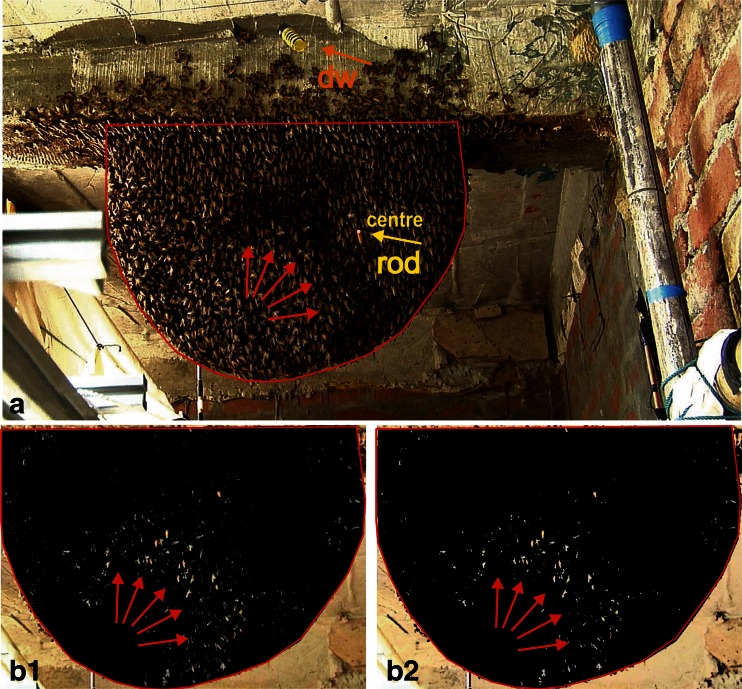
Detection of motion activity at the nest surface. **a** Original HD image of the nest with the dummy wasp (*dw*, see *arrow*) from 1 of 50 experimental sessions. The wooden rod for the LDV measurement (see *small yellow circle* marked by the *yellow arrow*) was fixed in the comb (by pushing it through), here at position *centre* (see Fig. 1). On the front plane of the rod, the original bright reflection spot of the laser beam is visible. The darker shadow-like areas in the plane of the nest (in front of the *red arrows*) indicate the presence of a shimmering wave which spread in the image hemi-cyclically from *bottom left* to *top right*. The *red line* around the nest gives the area of interest (AOI) in which motion detection was processed by image analysis technique. **b** Differential luminance images of the nest (AOI only), achieved by pixel-wise subtraction of frames (lum[*f*_*i*_] − lum[*f*_*i* − 1_]), revealing the motion-active surface bees at time *t*_*i*_ as bright pixel areas on the black background; **b**_**1**_ gives the differences in pixel luminance as *grey shades* (*black*: ∆lum = 0; *white*: ∆lum = 255), **b**_**2**_ shows the supra-threshold changes in luminance (*black*: ∆lum ≤ 5; *white*: ∆lum > 5) quantifying motion by segmented pixel areas (Kastberger et al. 2011a, b; compare Fig. 2b)

During shimmering, cohorts of bees in the surface layer of the nest flip their abdomens upwards in an angle which may exceed 90°. Synchronized and cascading processes emerge to a Mexican wave-like pattern. A light-emitting diode (LED) placed next to the comb was used to signal the start of the recording of the oscillations of the central comb. A video-recorded LED flash allowed synchronisation between video recording, image analysis and vibrometry. Each of the experimental sessions (*n*_s_ = 23) lasted for 6,292 frames (≡ 125.84 s) and included two phases: (a) an *arousal* phase, in which the colony had been stimulated by the presentation of the moving dummy wasp (Fig. 1), and (b) a subsequent *quiescent* phase, in which the dummy had been halted at its parking site at the upper left side of the nest (Fig. 1). The video films were re-formatted as sequences of jpg images using the Avid Media Composer editing software (Avid Technology, Inc.) in order to enable the pixel-based luminance analysis (ImagePro Plus, Media Cybernetics). Motion patterns of dummy (dw) and shimmering (sm) were quantified in terms of differences in pixel luminance (∆lum) between two sequential frames (Figs. 2 and 3; Supplementary Movie 1). Minor to no change in luminance values (∆lum ≤ 5) represented the ‘motionless’ state and were displayed as ‘black’ in the differential image. Change in luminance values of ∆lum > 5 signalled ‘movement’; they were segmented (Kastberger et al. 2011a, b, 2012, 2013) as ‘white’ spots and charted regarding the coordinates of their gravity points (shimmering: **x**_sm_, **y**_sm_) and their pixel areas per frame. The sum of white areas in a difference image of the nest surface was taken separately for dummy and shimmering (*A*_sm_) as the value for motion activity at the time the second sequential frame was captured (Fig. 2).

### Comparison of shimmering activity and comb vibration

**Fig. 4:**
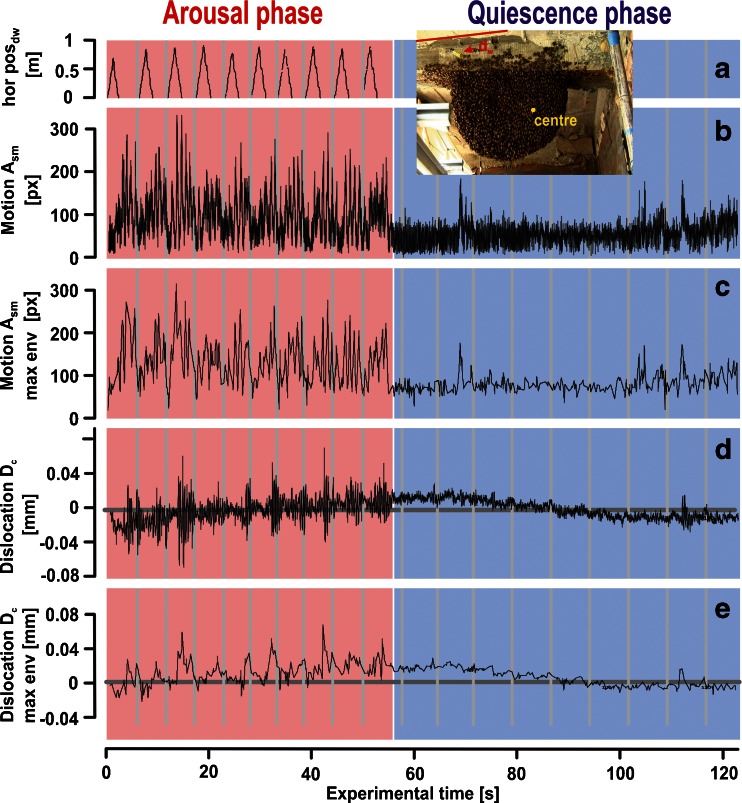
Motion at the nest surface and comb vibrations in the *arousal* (*pink background*) and *quiescence* (*blue background*) phases of a single experimental session. **a** The horizontal position of the dummy wasp (hor pos_dw_) over time during ten passes across and back in the front of the nest (which defined the *arousal* phase in the experiment) from position 0 m at the left side to position 1 m at the right side as depicted in the images of Figs. 1 and 3. **b** Time course of motion activity due to shimmering (*A*_sm_) at the surface of the experimental nest scaled in pixel area (see Fig. 3b_2_). The peak performance was caused by the movement of the dummy wasp (pictured in Fig. 2). **c** Envelope function of the maximal values of the motion activity *A*_sm_ shown in **b** (max env *A*_sm_, see command 1). **d** Vibrations scaled as dislocation (*D*_c_) over time of the central comb in millimetres, as assessed by LDV with the reflective end of the rod as target at the centre position of the nest (see *inset*). **e** Envelope function of the maximal values of the vibrations shown in **d** (max env *D*_c_, see command 1). The *grey vertical lines* in **a**–**e** define the interval limits in which correlations between shimmering and vibration activity (see Fig. 5) were calculated (see also Supplementary Movie 1)

For each experimental session, the motion patterns of the dummy (dw) and of shimmering (sm) at the nest surface were monitored together with the displacements of the central comb (*D*_c_) during the *arousal* (ar) and *quiescent* (qu) phases (Fig. 4; Supplementary Movie 1). The two ‘oscillatory’ signals, the shimmering activity and the comb vibration, exhibited different base (‘natural’) periods (*T*_sm_, *T*_c_) and base (‘natural’) frequencies (*f*_sm_, *f*_c_), respectively. Therefore, the momentary amplitudes of the signals (*A*_sm_, *A*_c_) could not be directly compared. We established a demodulation procedure by maxima enveloping by searching for peaks in the course of the amplitudes of both signals (^max^ *A*_sm_, ^max^ *A*_c_) within running intervals ([*f*_*i* − 2_, *f*_*i* + 2_], with *f*_*i*_ as the frame being examined) and by connecting the detected peaks by straight lines (Fig. 4c, e). The enveloped data of both signals (^env^ *A*_sm_, ^env^ *A*_c_) were then correlated for every time point *t*_*i*_ (at the frames *f*_*i*_) throughout both experimental phases. For the basic statistics (mean ± SE), the data were stepped according to the intervals of the dummy presentation cycles in the *arousal* phase and in 5-s intervals for the subsequent *quiescent* phase (see vertical intercept lines in Fig. 4).

## Results

### Shimmering activity dislocates mass in the bee curtain

The mass of a giant honeybee (*A. dorsata*) nest is determined by two assembly parts (Ruttner 1988; Kastberger 1999; Kastberger et al. 2011a, b), comb and bee curtain. The comb is built of wax cells with watery contents of larvae, honey and pollen. A single cell of the comb with a diameter of 6 mm and a length of 25 mm (values based on own observations) has a volume of 7.065 × 10^−7^ m^3^ which sums up for the comb with a double layer of cells (assuming a mean mass density of 0.80 kg/L due to the part of the wax) to 40 kg/m^2^ comb area.

The bee curtain consists of multiple layers of bees. In addition to the honey stores in specified comb areas in the genus *Apis* (Ruttner 1988), giant honeybees possess individual honey provisioning (own observations) as a social property which makes them particularly robust against short-term food shortages. Most of the shimmering-active curtain bees contain a mass of 100 mg or more of watery honey in their stomach (Oldroyd and Wongsiri 2006; Kastberger et al. 2011b), which accounts for the majority of the abdomen mass. Assuming that a single bee has a body weight of 150 mg, the bee curtain of a nest of 50,000 bees would weight approximately 7.5 kg. Based on these estimates, large giant honeybee nests could achieve a mass of more than *M*_nest_ = 50 kg (Ruttner 1988).

Colony members as honey storers become much heavier in their abdomens compared to their counterbalancing heads. This aspect is particularly important for the shimmering action, whereby surface bees flip their abdomens (abd) up at angles ranging from 20° to 120°, depending on their *arousal* level (Kastberger et al. 2011a, b). From a physics viewpoint, each bee acts here as a torsion pendulum (Kastberger et al. 2011b) with an acentric axis and two disparate masses on both sides of the rotation centre, the upward head and the downward abdomen. Abdomen flips cause a single oscillation or multiple oscillations of the mass of the abdomen in a curved motion. Mass inertia produces a reaction force, which initially presses the individual bee with the thorax towards the nest, but consecutively, the thrust of the pendulum mass initiates a centrifugal force which is directed away from the comb (see a movie of an abdomen-flipping model bee in Kastberger et al. 2011b). Both motion components affect the *z*-direction, which can be defined regarding the inertial system of the nest as towards and away from the comb (which defines the *z*-direction). In this way, a single bee would provoke a centrifugal force of *F*_*z*_ = 1.266 mN (Eq. 1), assuming an abdomen mass [*M*_abd_] of 100 mg of honey and the respective flipping properties (angle [⊖_abd_], 90°; duration [∆*t*_abd_], 80 ms; radius of abdomen length [*l*_abd_], 10 mm):

1 $$ {F}_z={M}_{\mathrm{abd}}\times {\omega}_{\mathrm{abd}}{}^2\times {l}_{\mathrm{abd}},\kern0.5em \mathrm{with}\kern0.5em {\omega}_{\mathrm{abd}}={\ominus}_{\mathrm{abd}}/\varDelta {t}_{\mathrm{abd}}\kern0.5em \mathrm{as}\kern0.5em \mathrm{angular}\kern0.5em \mathrm{velocity} $$

In the collective action of shimmering (Seeley et al. 1982; Kastberger et al. 2011a, b) in which hundreds of surface bees are synchronised and cascading within a fraction of a second, the centrifugal forces of the shimmering-active surface bees may total up to more than 1 N and pull the subsurface layers of the bee curtain away from the comb (Kastberger et al. 2011a). For an *A. dorsata* nest, this magnitude of force is powerful enough to shift a significant mass of the bee nest for a fraction of a second to an observable extent. Under this mechanical condition, a giant honeybee nest might be considered as a driven physical pendulum (see Online Resource 1). This is even enhanced by the fact that the mechanical perturbation caused by the concerted wave-like patterns of abdominal flips occurs asymmetrically at only this side of the bee curtain at which the surface bees had been threatened by the visual cue.

### Shimmering activity provokes comb vibrations

**Fig. 5:**
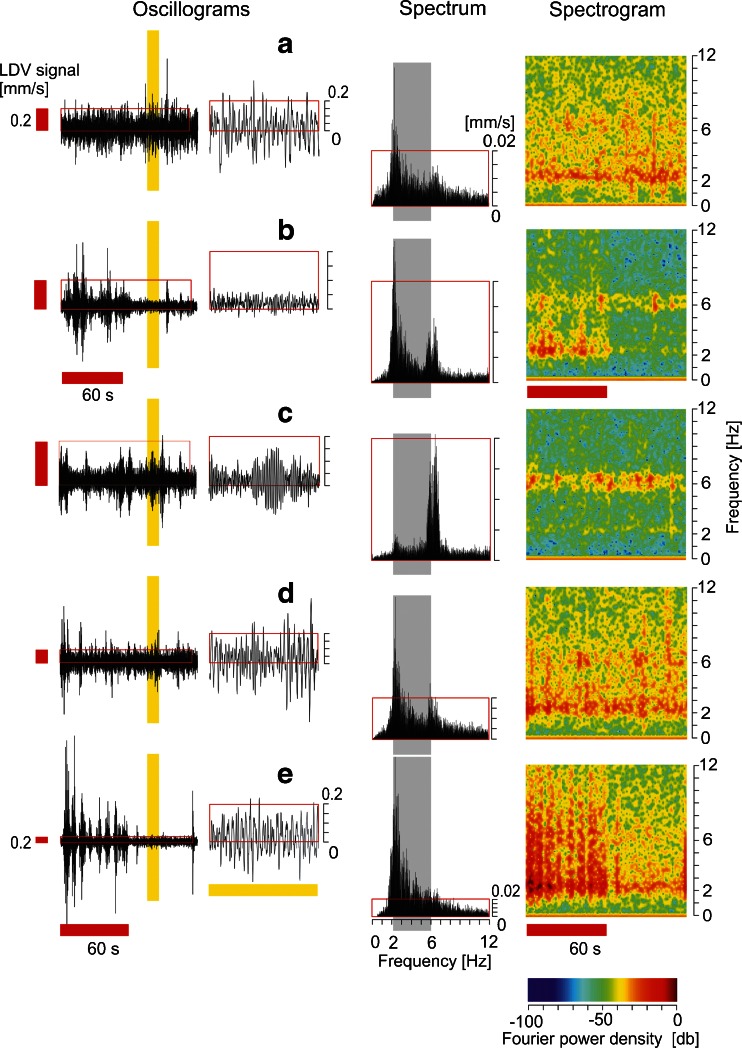
LDV signals of the comb under different experimental conditions. **a** Quiescent conditions after the mass flight activity, LDV signals recorded at position S1 (Fig. 1); the dummy wasp was at its parking site. **b** The dummy wasp was presented for 60 s (see time scale as *red horizontal bar*), LDV measurement at position S1. **c** Mass flight activity without the presentation of the dummy wasp, LDV measurement at position S1. **d** Quiescent conditions without the presentation of the dummy wasp, LDV signals recorded at position S2 (Fig. 1). **e** The dummy wasp was presented for 60 s (see time scale as *red horizontal bar*), LDV signals recorded at position S2. ‘Oscillograms’ panels, *left-side panels* refer to LDV oscillograms (ordinate [*A*]: velocity in millimetres per second) of the whole experimental session (abscissa, 125 s), *right-side panels* refer to 10 s (from 80 to 90 s of the original session, as documented by the position and width of the *vertical yellow bar*). ‘Spectrum’ panels refer to frequency spectra of those sessions documented in the 125-s oscillograms; abscissa: frequency range in hertz; ordinate: amplitude parameter (velocity in millimetres per second). ‘Spectrogram’ panels: abscissa: experimental time; ordinate, frequency; *z*-axis, PSD from −100 to 0 db; PSD = 10 log10 ABS (*A*_*i*_/*A*_0_)^2^), with *A* [velocity in millimetres per second] as the amplitude of the Fourier coefficients; see scale of colour codes

**Fig. 6:**
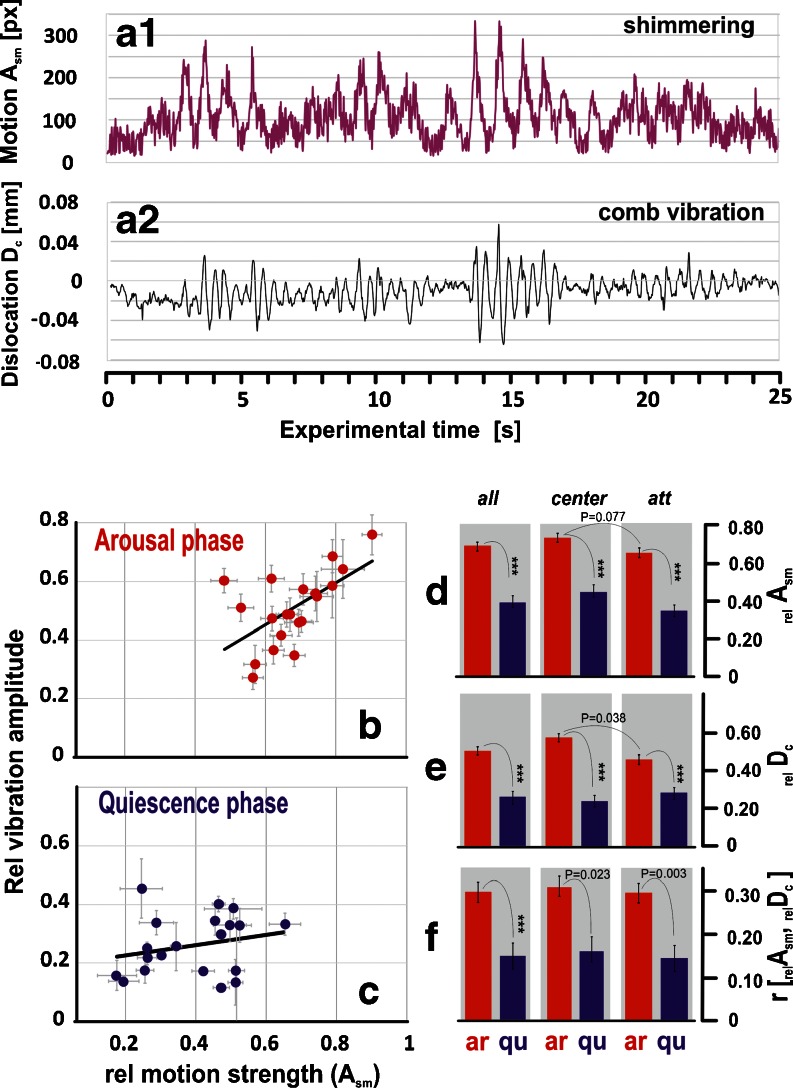
Correlation of the comb vibrations and the shimmering activity. For measurement at the position *centre*, samples of motion activity at the nest surface are shown during shimmering (**a**_**1**_) and the corresponding vibrations at the comb (**a**_**2**_). **b**, **c** Abscissa, motion strength (_rel_ *s*_m_); ordinate, dislocation per 0.02 s: _rel_ *D*_c_ of vibrometer signals in the *arousal* (ar, *red*) and *quiescence* (qu, *blue*) phases; for every experimental session (*n*_s_ = 21), the envelope data of comb vibrations and shimmering motions per frame were correlated. *Full circles* arithmetical means, *horizontal and vertical bars* SEMs; regression functions of the mean values: *arousal phase* (_rel_ *D*_c_ = 0.080 + 0.676*s*_m_; *R* ^2^ = 0.413); *quiescence phase* (_rel_ *D*_c_ = 0.192 + 0.176*s*_m_; *R* ^2^ = 0.0549). **d**–**f** Comparison between *arousal* and *quiescent* phases: motion strength at the nest surface _rel_ *A*_sm_ (**d**), comb vibration as relative dislocation _rel_ *D*_c_ (**e**) and correlation coefficients *r* [_rel_ *A*_sm_, _rel_ *D*_c_] (**f**) of the means (*n*_s_ = 21) at all positions and at the positions *centre* (*n*_s_ = 9) and *att* (*n*_s_ = 12); *vertical black bars* indicate SEMs; statistics: ***P < <0.01 (*t* test)

In the experiments presented in this paper, two phases of the nest are distinguished (Fig. 4; see Online Resource 2/Movie 1): the *arousal* phase, in which the dummy wasp was presented in subsequent cycles provoking shimmering waves (Fig. 4a), and the *quiescent* phase, in which the dummy stayed positioned at the parking site (see the ‘Material and methods’ section). Motion activity at the nest surface was low in *quiescent* phases (Figs. 4, 5 and 6), but rose significantly during *arousal* in general, such as during mass flight activity (Kastberger et al. 1996) with diffuse, unsynchronized locomotion, or in shimmering activity, in which the abdomens predominantly of surface bees display coordinated, repetitive and, therefore, portioned motions of patterns over time (Kastberger et al. 2011a, b; Fig. 4b). All these motions of the curtain bees provoke mass shifts which give rise to associated vibrations at the comb (Fig. 4d).

Generally, shimmering waves are produced by collective decision-making of the surface bees, whereby the natural repetition rate and the motion strength of the shimmering waves (Kastberger et al. 2008, 2011a, b) depend on the *arousal* level of the participating surface bees. We observed waves at a repetition rate of wps_sm_ = 0.957 ± 0.030 Hz (*N*_wav_ = 1,287 waves in 1,361 s recording time, with wps as waves per second). The sequences of comb vibrations showed, particularly in the *arousal* phases, a rhythm in their modulation similar to that of the associated shimmering waves (Fig. 4b, d). This coincidence convincingly indicates that the comb oscillations were incited through the driving force of the shimmering process. The sinusoid oscillations at the comb had base frequencies of pps_c_ = 2.156 ± 0.042 Hz (*N*_p_ = 2,887 periods in 1,341 s; with pps as periods per second) which are more than double the average repetition rate of shimmering waves.

In Fig. 5, five experimental sessions exemplify the comb vibrations displayed by oscillograms, spectrograms and frequency spectra of original LDV velocity data (see the ‘Material and methods’ section). The first session (Fig. 5a) refers to the basically quiescent condition at position S1 (Fig. 1) near the attachment zone. In this session, small shimmering waves happened which had been released by colony-intrinsic reasons. Two frequency bands are documented in the comb vibration, one at the basic frequency of slightly more than 2 Hz, as calculated previously, and a second band at roughly 6 Hz.

The second session (Fig. 5b) refers to the main experimental condition investigated in this paper in which the presentation of the dummy wasp gave rise to shimmering waves in the *arousal* phase, while in the post-presentation phase, the colony returned to *quiescence*. In this session, comb oscillation is displayed on both frequency bands, whereas it becomes clear that the shimmering waves provoked oscillations at the lower band, while in the post-presentation phase, these lower band activities terminated while the small oscillations at the 6-Hz band still continued. There are two reasons why these comb oscillations provoked by shimmering waves (Fig. 5b) are much smaller than those under Fig. 5e. First, the shimmering waves happened mostly at nest regions other than at position S1 (which was not in the centre of the shimmering patterns), and second, the position S1 was near the attachment zone of the nest.

The third session (Fig. 5c) refers to a period of mass flight activity in which the colony turns into a general state of arousal (Kastberger et al. 1996). Here, locomotor behaviour at the nest surface is combined with heavy flight activity, whereby bees start from the outer layers of the bee curtain and return to the nest some minutes later. In this state, the colony is in turmoil, revealing a characteristic diffuse mechanical activity of locomotor actions and small shimmering waves provoked by returning bees (Kastberger et al. 2011a). The striking difference here to all other samples of Fig. 5 is that the comb oscillations are dominantly represented at the frequency band at 6 Hz. This preference could be characteristic for the driving force as recruited under mass flight activity. However, the velocities of the comb oscillations are here at a low level compared to those at position S3 (Fig. 5d, e).

The fourth sample session (Fig. 5d) refers again to quiescent conditions without the presentation of the dummy wasp as documented under Fig. 5a. Also, here, small ‘spontaneous’ shimmering waves happened and the comb oscillations are reflected at both frequency bands. However, the vibrations reveal a much higher intensity (as displayed in the oscillograms and in the spectrum), which is obviously caused by the greater distance of position S3 (Fig. 1) from the attachment zone of the nest.

Lastly, session 5 (Fig. 5e) refers to position S2 and to the sequence of two phases, the arousal phase, in which shimmering waves were elicited by the presentation of the dummy wasp, and the subsequent quiescence phase, without the presence of the dummy. The shimmering-provoked comb oscillations reveal a broad frequency band with a clear peak at slightly above 2 Hz, while in the quiescent phase, both frequency bands were better contrasted (compare Fig. 5d). The shimmering-induced oscillations were quite intense, which is due to the fact that the measurement was taken from position S2 which was located near the main path of the shimmering waves and near the lower rim of the nest.

These vibration spectra of the compound physical system of the nest reveal two strikingly constant frequency bands: the low band of slightly above 2 Hz can be plausibly interpreted as the *natural* frequency of this compound system consisting of the comb and bee curtain because it is factually independent regarding the frequency performance from the driving forces, which may be provoked by shimmering waves (Figs. 4 and 5b, e) or by the mass flight activity (Fig. 5c). Both arousal states chosen differ in their time profiles of the driving forces: shimmering waves are rhythmic and strong and the mass flight activity provides more stochastic and much weaker influence. Nevertheless, in the frequency spectra, two prominent bands are visible, at >2 and 6 Hz, which is slightly more than double of the base frequency (Fig. 5a–e).

The samples in Fig. 5 let us assume that the driving forces of shimmering waves and mass flight activity feed their energy into different frequency bands: shimmering waves regard the low band of slightly above 2 Hz and mass flight activity regards the higher band at 6 Hz. The fact that both frequency bands can be detected under both, quiescent and arousal, conditions, though at different intensities and at different locations, supports the view that the compound system of the comb and bee curtain functions as an elastic plate oscillator.

### Correlation of comb oscillations with shimmering waves

Both periodic processes, the comb oscillations and the shimmering waves, differed in their basic frequencies (Fig. 4). For correlation, time-dependent power levels of both signals were compared throughout the experimental sessions frame by frame, estimated by peak enveloping (see Fig. 4), by collecting the arithmetical means ± SE of the strength levels of the shimmering signals (*A*_sm_) and of the vibration (*A*_c_) in stepped intervals (∆*t* = 0.02, FPS = 50 Hz). The amplitude of the vibration between the two time points is further on termed as dislocation (*D*_c_).

The two enveloped data sets (*n*_exp_ = 21 experiments; *n*_int_ = 132,321 time intervals of 0.02 s) correlated positively (for regression functions, see Fig. 6). The comb oscillations showed under *arousal* conditions due to shimmering waves significantly (*P* < 0.001, Student test) larger dislocations than under *quiescent* conditions (*arousal*: *D*_c_ = 7.230 ± 0.573 μm per 0.02 s frame interval, which equals to *D*_c_ = 0.3099 ± 0.0286 mm/s, *n*_s_ = 388 dummy wasp sessions; Fig. 6a; *quiescence*: *D*_c_ = 3.489 ± 0.283 μm per 0.02 s, which equals to *D*_c_ = 0.1745 ± 0.142 mm/s, *n*_s_ = 150 post-*arousal* sessions; Fig. 6b).

### Spatial characteristics of the physical excitability of the comb

The two comb positions (*centre* and *att*; Figs. 1 and 6) represent, under the preposition of a physical pendulum (see Online Resource 1 and the ‘Discussion’ section), two sample distances taken from the pivotal axis. The basic frequencies in the *arousal* phase were slightly above 2 Hz (position *centre*, 2.112 ± 0.063 Hz; *n*_p_ = 1,779 oscillation cycles in 842 s; position *att*, 2.225 ± 0.034 Hz; *n*_p_ = 1,108 periods in 499 s) and did not significantly differ (*P* = 0.131, Student test; *n*_exp_ = 21) between both positions. The enveloped amplitudes of oscillations (^env^ *A*_sm_, ^env^ *D*_c_; see the ‘Material and methods’ section) were related per session to the maximal value to compensate for differences in the recording conditions in the experimental sessions. The dislocations of comb vibration (_rel_ *D*_c_; see Eqs. 2a, 2b, and 2c) under *arousal* were markedly higher at position *centre* (*n*_exp_ = 9) than at position *att* (*n*_exp_ = 11; *P* = 0.059, Student test; Fig. 6d):

2a $$ {D}_{\mathrm{c}}\left[{t}_{i-1},{t}_i\right]=\varDelta {A}_{\mathrm{c}}/(0.02)\left[\mathrm{mm}/\mathrm{s}\right]\kern0.5em \mathrm{with}\kern0.5em \varDelta {A}_{\mathrm{c}}=\left({A}_{\mathrm{c}}\left[{t}_i\right]-{A}_{\mathrm{c}}\left[{t}_{i-1}\right]\right)\kern0.5em \mathrm{for}\kern0.5em \mathrm{every}\kern0.5em \mathrm{inter}-\mathrm{frame}\kern0.5em \mathrm{inter}\mathrm{val}\kern0.5em \varDelta {t}_{ff}=t\left[{f}_i\right]-t\left[{f}_{i-1}\right]\kern0.5em \mathrm{at}\kern0.5em 50\;\mathrm{fp} $$

2b $$ {}_{\mathrm{rel}}{D}_{\mathrm{c}}={D}_{\mathrm{c}}\left[{t}_{i-1},{t}_i\right]/ \max {D}_{\mathrm{c}}\left[{t}_{i-1},{t}_i\right] $$

2c $$ {A}_{\mathrm{c}}\left[{t}_{i-1},{t}_i\right]=\varDelta {A}_{\mathrm{c}}/\left({0.02}^2\right)\left[\mathrm{mm}/{\mathrm{s}}^2\right] $$

The shimmering activities in the two series of experiments concerning the measurement positions *centre* and *att* had similar strength levels (*P* = 0.077, Student test; Fig. 5c), which allows comparison of the associated dislocations. This consistency is also expressed by the *arousal* factor *F*_ar_ (Eq. 3; *centre*: *F*_ar_ = 1.64; *att*: *F*_ar_ = 2.05; *P* = 0.83, chi-square test) which denotes a similar proportion between the motion levels in the subsequent experimental phases of *arousal* (ar) and *quiescence* (qu):

3 $$ {F}_{\mathrm{ar}}={}_{{}_{\mathrm{rel}}}{A}_{{}_{\mathrm{sm}}}\left[\mathrm{ar}\right]/{}_{\mathrm{rel}}{A}_{\mathrm{sm}}\left[\mathrm{qu}\right] $$

As expected, the correlation coefficients between _rel_ *D*_c_ values of comb vibration and the _rel_ *A*_m_ values of motion strength at the nest surface yielded higher (*P* < 0.05, Student test) magnitudes under *arousal* than under *quiescence* and had also similar proportions regarding both measurement positions (Fig. 6e).

### Assessment of cycle-based dislocation and acceleration spectra of comb vibrations

To compensate for obvious noise effects (see Online Resource 1) and to prove perspectives for potential communication goals, the dislocation and acceleration spectra have to be considered regarding the basic (‘natural’) period of the vibration cycles of the comb (*T*_c_ = 1 / *f*_c_) under shimmering (*arousal*) conditions which was assessed as pps_c_ = 2.156 ± 0.042 Hz (see previous section). For that, we determined the peaks and sinks of the time integral of the LDV signal with an automated method according to the algorithm of command 1:

Command 1

- IF (*A*_*i*_ − *A*_*i* − 3_) > *A*_threshold_ AND (*A*_*i*_ − *A*_*i* + 3_) > *A*_threshold_ THEN PEAK [*f*_*i*_] = TRUE
- IF (*A*_*i* − 3_ − *A*_*i*_) > *A*_threshold_ AND (*A*_*i* + 3_ − *A*_*i*_) > *A*_threshold_ THEN SINK [*f*_*i*_] = TRUE

checking every frame *f*_*i*_ for exceeding the respective threshold conditions with A_threshold_ = 5 μm.

This procedure factually introduced a digital low-pass filter (with *T*_c_ > 20*∆*t*_ff_) and documented the dynamics at the basic frequency of the comb. It allowed the assessment of dislocation (or of acceleration) values per oscillation cycle of the comb and utilized the |∆*A*_p,s_| values between a peak and the subsequent sink (or between a sink and the subsequent peak) for calculating the respective dislocations *D*_vc_ within vibration cycles (Eq. 4):

4 $$ {D}_{\mathrm{vc}}=\left|\varDelta {A}_{\mathrm{p},\mathrm{s}}\right|/\varDelta {t}_{\mathrm{c}}\kern0.5em \mathrm{and}\kern0.5em {a}_{\mathrm{c}}=\left|\varDelta {A}_{\mathrm{p},\mathrm{s}}\right|/\varDelta {t}_{\mathrm{c}}{}^2\kern0.5em \mathrm{with}\kern0.5em \left|\varDelta {A}_{\mathrm{p},\mathrm{s}}\right|=\left|{A}_{\mathrm{PEAK}}-{A}_{\mathrm{SINK}}\right|\kern0.5em \mathrm{and}\kern0.5em \varDelta {t}_{\mathrm{vc}}={T}_{\mathrm{c}}=1/{f}_{\mathrm{c}};\kern0.5em {f}_{\mathrm{c}}=2.114\;\mathrm{Hz}\kern0.5em \left(\mathrm{see}\ \mathrm{the}\ \mathrm{previous}\ \mathrm{paragraphs}\right) $$

**Fig. 7:**
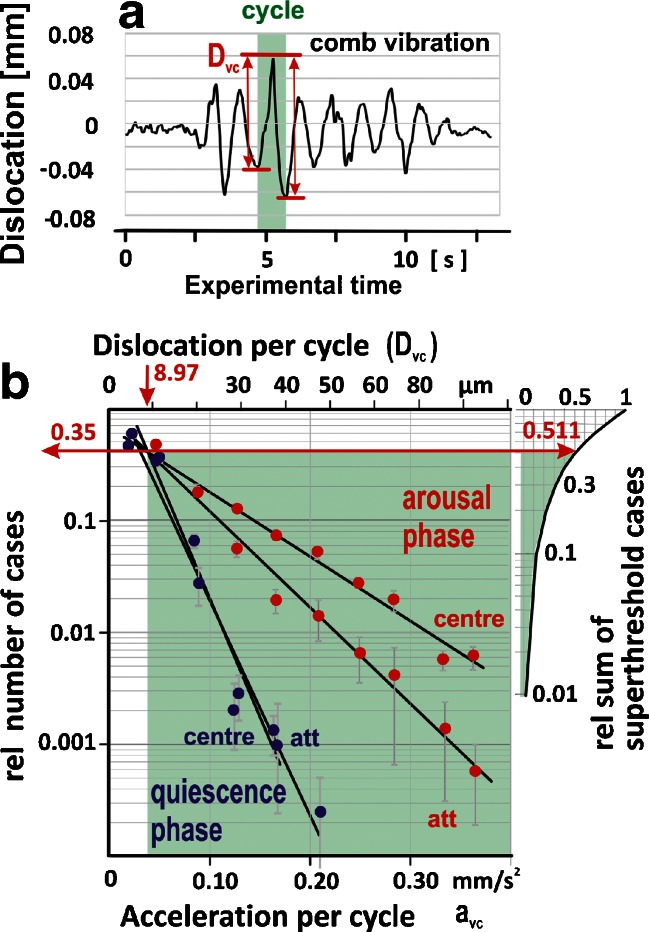
Cycle-based dislocation spectra at the comb in the experimental *A. dorsata* nest as provoked by shimmering waves. **a** Definition of dislocation (*D*_vc_) per cycle of comb vibration (*f*_vc_ = 2.114 Hz; *T*_vc_ = 0.473 s) as the distance [in micrometres] between a sink and the successive peak of the time-integrated DLV signal or between a peak and the successive sink. **b** Spectra of accelerations (*a*_vc_) and dislocations (*D*_vc_) of the comb of the experimental *A. dorsata* nest in the *arousal* and *quiescence* phases of experiments: ordinate, relative numbers _rel_ *N*_vc_ of vibration cycle (*vc*) intervals of the respective class value (*D*_vc_); abscissa, ten classes of dislocations *D*_vc_ per cycle of comb vibration. *Full circles* and *vertical bars* give the means ± SEMs. *Black lines* regression functions of the means: *y* = *ce* ^*xd*^; with *y* = _rel_ *N*_vc_; *x* = *D*_vc_; *R* ^2^ > 0.94; *N*_vc_ = 12,686. The *green area* refers to supra-threshold (*D*_vc_ > 8.97 μm per *T*_vc_, after Sandeman et al. 1996) dislocation amplitudes which made up 51.1 % of cases (accessory diagram on the *right*)

The resulting spectra of displacement and acceleration reveal (Fig. 7) that, under *arousal* conditions, 51.08 % of vibration cycles of comb oscillations exceeds the sensory threshold of honeybees (calculated after Sandeman et al. 1996). The spectra of Fig. 7 strongly differ (*P* ≪ 0.01, Student test) for both positions under *arousal* (*centre*: *n*_vc_ = 3,160; *att*: *n*_vc_ = 3,416) and between *arousal* and *quiescence* (*centre*: *n*_vc_ = 3,215; *att*: *n*_vc_ = 2,895), but not under *quiescence*. The data suggest that the spectra are primarily shaped by differences in amplitudes of the vibrations and not by different noise levels (see Supplementary Text 1).

## Discussion

### Communicative impact of substrate-borne vibrations

The shimmering process in giant honeybees (Kastberger et al. 2008, 2011a, 2012, 2013) exemplifies the principle that fast and accurate spreading, gathering and sharing of information entails the success of group-living species (Evans et al. 2007). In an *A. dorsata* colony, the collective of curtain bees form for the purpose of repelling enemies (Kastberger et al. 2008, 2011a, b) adaptive patterns in the visual, pheromone and mechanical domains. Visual cues trigger and flexibly adjust the Mexican wave-like shimmering process (Kastberger et al. 2010, 2011a, 2013; Schmelzer and Kastberger 2009). Nasonov scenting (Kastberger et al. 1996) modulates the coherence and strength of the shimmering process. Mechanical traits, synchronised and cascading as displayed by the waves in the visual domain, are produced by the abdomen-lifting actions (Kastberger et al. 2011a, b) of bees primarily in the surface layer. These mechanical activities cause mass shifts in the nest which take place within fractions of a second (Kastberger et al. 2011a, 2012, 2013) and are asymmetrical regarding the nest geometry, as shimmering primarily concerns the threatened nest side. However, the forces associated with these mass shifts affect the whole nest and thus also the centrally positioned comb. The present paper questions here whether the shimmering-active surface bees do have the capacity to establish comb vibrations which are supra-threshold (Sandeman et al. 1996) cues to provide intra-colony information for the entirety of the nest mates (Figs. 1, 4, 5, and 6).

Use of substrate-borne vibrations for communicative goals is well known in arthropods. It has been described in sap-sucking bugs when signalling presence, attraction, alarm or defence between group members (Cocroft 1996; Cocroft and Rodriguez 2005; Hartbauer 2010) and also in termites (Evans et al. 2007), sawflies (Carne 1962) and caterpillars (Claridge 1985; DeVries et al. 1993; Cocroft and Rodriguez 2005; Yack et al. 2001; Fletcher et al. 2006). Social hymenoptera use vibration of substrates as signals, particularly for recruiting foragers (ants: Roces et al. 1993; Roces and Tautz 2001; dancing honey bees: Michelsen et al. 1986; Kirchner 1993; Tautz et al. 1996; Nieh and Tautz 2000; Hrncir et al. 2006), whereas the individual insects’ pounding, beating or knocking on a substrate are utilized as the sources of mechanical energy.

Honeybees contrast herein with other vibration-producing arthropods insofar as they utilise the comb as a substrate, which is built by the community itself, to which they directly cling for transmitting their communicative signals (Nieh and Tautz 2000). However, the shimmering process of giant honeybees and its relationship to comb vibration is different to the mechanical activities in the Western honeybees (Nieh and Tautz 2000). In giant honeybees, the shimmering-active individuals are positioned on the surface of the bee curtain. They have several layers of colony members underneath, which separate them from direct access to the comb and dampen the direct energy transfer from surface to comb. Nevertheless, the data presented in this paper provide evidence that the shimmering action yields sufficient power to drive the comb into measurable magnitude of vibration (Figs. 4, 5, 6, and 7).

### Testing the colony-intrinsic information hypothesis in giant honeybees

In this paper, we provide first evidence that shimmering behaviour (Kastberger et al. 2011a, b, 2012, 2013) of giant honeybees may essentially contribute to intra-colonial communication. The findings may support the colony-intrinsic information hypothesis (Kastberger et al. 2008) which assumes that shimmering has the potential to signal the momentary state of colony defence to the entire community, directing information to all curtain bees, even to those which have not actively participated in the wave. The concerted power of hundreds of shimmering-active surface bees conveys information through the multiple curtain layers. The centrally located comb in a giant honeybee nest is here the only element which does have the capacity of transmitting mechanical energy across the nest, in particular from the threatened to the contra-lateral, non-threatened side. This colony-intrinsic information hypothesis also addresses that both behaviours, shimmering and comb-building, may have customized themselves in relation to each other in the course of evolution, resulting in a cross-linked fine-tuning of their physical properties. Giant honeybees could herein serve as a prominent example for the performance of information transfer among colony members (summarised for arthropods by Evans et al. 2007 and more generally by Alcock 2005).

However, there is still an essential question which begs for an answer in future work: Would this sort of mechanoreceptive stimulation across the comb as provoked in shimmering waves actually bias or even benefit curtain bees on the non-threatened side? Although the data of the present paper leave this aspect untouched and cannot clarify this, we nevertheless back up the possibility and importance of comb-borne vibrations for colony-intrinsic communication by enlisting relevant physical properties of giant honeybee nests which are specifically affected by shimmering.

### Mechanical requirements enabling colony-intrinsic communication

Vibrations of the comb during shimmering exhibited a basic frequency of slightly over 2 Hz. They were excited by the natural repetition rate of the shimmering waves at slightly <1 Hz. This finding demonstrates independence between both frequencies which is an attribute for an obvious property of resonance of the comb. It is the nature of resonant systems (Nave 2001) to respond to external periodic influences, whereas the driven system tends to respond to frequency components of the external forces, which are close to and preferably slightly below the respective frequency bands (Nave 2001), and also to ultimately settle down to a performance determined by the driving force. The empirical data prove here the comb as an oscillator (Figs. 4, 5, and 6) and the shimmering activity as the force which drives the comb at half of its basic frequency band. Furthermore, the comb vibrations approached zero in a short exponentially decaying sinusoid function (Fig. 4d), which expresses under-damping of a potentially harmonic oscillator (Nave 2001). In this respect, it is quite remarkable that the diffuse forces produced during mass flight activity (Kastberger et al. 1996) affect the comb at its possibly first harmonic component at 6 Hz. These physical properties of the comb have been exemplified in the experimental nest but may happen in similar performance (possibly at slightly different frequency bands) in other giant honeybee nests of different sizes or ages.

**Fig. 8:**
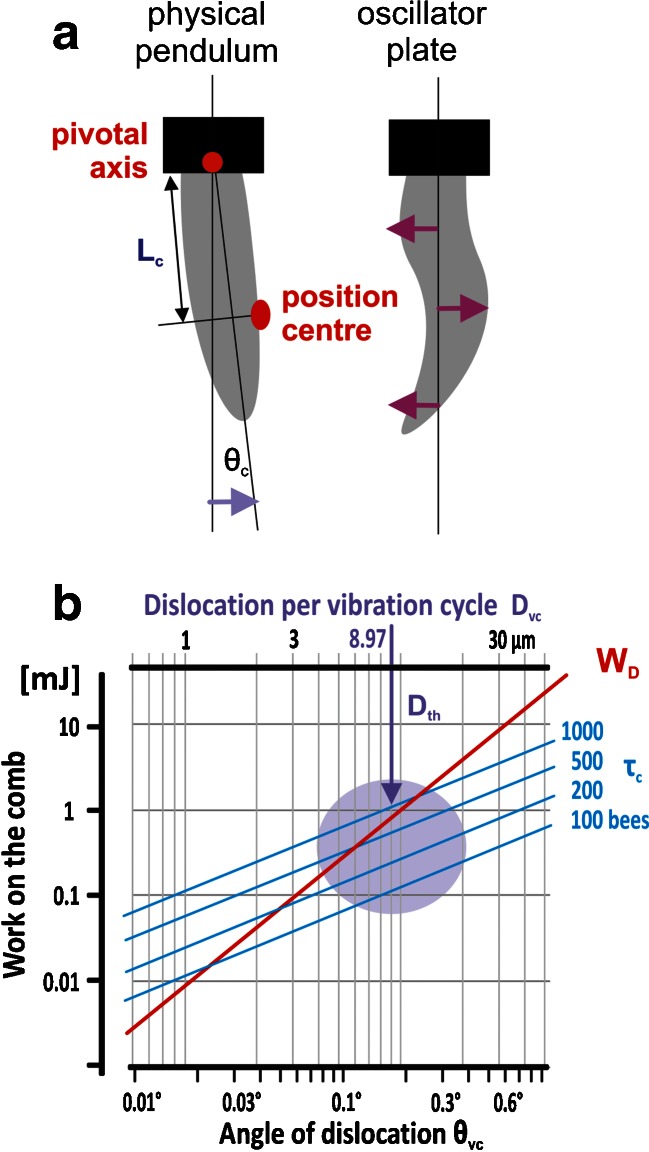
How empirical data match the pendulum dislocation theory in the experimental *A. dorsata* nest. **a** Sketches of the comb of an *A. dorsata* nest as a physical pendulum and as an oscillator plate. Dislocation angle (⊖_c_) of the comb, distance between the pivotal axis and the position *centre* as a substitute for the pendulum length *L*_c_. **b** Lookup table of work on the comb against dislocation length (*D*_vc_) and dislocation angle (⊖_vc_) per cycle of the comb vibration at position *centre* (*L*_c_ = 30 cm). The *red line* gives the work *W*_D_ (Eq. 2b) which considers the dislocation and the acceleration in the half cycle of a vibration (*T*_vc_/2); the *blue lines* give the torque (Eq. 3) estimated under four conditions of *F*_sh_ when 100–1,000 surface bees act synchronized but asymmetrically on one side of the comb during shimmering. The *vertical arrow D*_th_ gives the threshold dislocation estimated after Sandeman et al. (1996) for a vibration of 2 Hz. It is illustrated that both work values match for the threshold dislocation in the small area denoted by the *violet full circle*

These mechanical features of the vibrations, measured at the comb, and the driving forces of shimmering waves or of mass flight activity (Fig. 5) lead to two different, not exclusive, views of driven oscillator models explaining the compound physical system of an *A. dorsata* nest in general: it may behave as a physical pendulum or/and as an elastic mechanical plate (Fig. 8a). The question into which model was most fitting to the empirical data would have significance on the extent to which mechanical signals could be transmitted across the bee curtain.

#### Matching the empirical data with the physical pendulum theory

A typical giant honeybee (*A. dorsata*) nest, alike the experimental nest used for this investigation, would display a mass of *M*_c_ > 30 kg with a vertical extension of *L*_c_ > 50 cm. If excited as a physical pendulum with a typical torque force of shimmering of *F*_sh_ < 1 N (Eqs. 1 and S3b), it may swing around the pivotal axis at its specific frequency (*ω*_c_ ≈ 2 Hz) with small angular displacements (⊖_c_ < 0.5°) and a moment of inertia *I*_c_ (Eqs. S1a and S3s) which ranges between *I*_c_ = 0.55 kg/m^2^ (*M*_c_ = 30 kg) and *I*_c_ = 0.90 kg/m^2^ (*M*_c_ = 50 kg).

In the experimental nest, we proved the coincidence between the empirical data and the physical pendulum model in the work domain twofold (Fig. 8b): under a linear approximation (*W*_D_, Eq. S2) and under the rotational (*τ*_c_, Eq. S3) concept. The work *W*_D_ on the mass of the comb during one half of a vibration cycle is defined (Eq. S2b) by the product of the dislocating force *F*_D_ in the model nest (*M*_c_ = 50 kg; *L*_c_ = 30 cm; measurement position *centre*) and the empirically determined measures of its basic pendulum cycle (Eq. S2, with *T*_c_ = 0.4638 s; see Fig. 4b, d) under small dislocations (*D*_c_ = *L*_c_ × sin ⊖_c_ < 10 μm).

The rotational aspect of the pendulum model is quantified by the torque (*τ*_c_) of the comb (Eqs. S3 and S5) and considers the empirically estimated driving force *F*_sh_ (which is set up by the cohorts of shimmering-active bees at the surface of the nest; Eq. 5), the length of the pendulum rod (with *L*_c_ = 30 cm, as represented by the distance between the pivotal axis and the measurement position *centre*) and the empirically determined angle of the natural vibration cycle ⊖_c_:

5 $$ {\tau}_{\mathrm{c}}={L}_{\mathrm{c}}{F}_{\mathrm{sh}} \sin {\ominus}_{\mathrm{c}}\left[\mathrm{Joule}\right] $$

As result, the torque lines (Fig. 8b) of a variable number of shimmering-active bees (*N*_bees_ = 100–1,000) show gentler slopes than the *W*_D_ curve. We assume that the best correspondence of both work models (*W*_D_, *τ*_c_) is given at the crossing points of the functions which occurred at dislocations of ⊖_c_ < 0.2°, correspondingly *D*_c_ < 12 μm, per vibration cycle. Remarkably, this crucial dislocation values coincide with that of the sensory threshold of honeybees (*D*_th_ = 8.97 μm) as estimated after Sandeman et al. (1996) for 2 Hz and refer, theoretically, to a driving force produced by a shimmering wave of *N*_bee_ = 500–1,000 active bees (Fig. 8b).

#### Requirements for acceptance of the pendulum hypothesis

This match of theoretical and empirical data in the work domain supports the view that a giant honeybee nest shows the performance of a physical pendulum (see Supplementary Text 1), with the shimmering activity of surface bees as its driving force. However, for the acceptance of this (*shimmering-drives-the-nest-as-a-*) *pendulum* hypothesis, the empirical data of the comb have to meet the further three predictions: First, the natural frequency [*ω*_c_] of the real comb should match with the mathematically expected natural frequency of a physical pendulum of similar shape (*M*_c_, *L*_c_); second, the real comb should show the same natural frequency along its full ‘rod’ length [*L*_c_]; and third, the amplitudes of the oscillations (⊖_c_, *D*_c_) should show a gradient according to their distance from the pivotal axis.

The measurements confirm these expectations: First, the basic (‘natural’) period of the comb (*T*_c_ = 0.4682 ± 0.0106 s; *n*_s_ = 23 sessions) of the experimental nest (*M*_c_ = 40 kg, *I*_c_ = 0.7 kg/m^2^) under the regime of shimmering waves match the theoretical predictions (see Eq. S1a). Interestingly, under mass flight activity, the vibrations occur at 6 Hz which demonstrates that the comb also functions as an elastic plate. Second, the basic periods of the comb vibration differed only slightly between the main measurement positions (*centre*: *T*_c_ = 0.4798 s; *att*: *T*_c_ = 0.4503 s; *P* = 0.1312, *t* test). And third, the data document a gradient in the dislocation amplitude *D*_c_ from the attachment zone down to the lower rim in two aspects: the comb displacements _rel_ *D*_c_ were larger at position *centre* than at position *att* (Fig. 6d; *P* = 0.038, Eqs. 2a, 2b, and 2c) and the occurrence of larger displacements was higher at position *centre* (*P* < 0.001, *t* test) than at position *att*. These latter results were achieved in the frame-based (Fig. S1c–e) and cycle-based (Fig. 8b) filtered data of comb vibrations and are also documented in the spectra (Fig. 5).

#### Matching the empirical data with the alternative elastic mechanical plate model

The second approach to explain the swinging comb of a giant honeybee nest under the regime of shimmering is to compare it with a plane elastic structure oscillating at its natural resonance frequency. This view is obvious because the centrally positioned comb is built of wax stiffed by the watery cell contents of honey or jelly, which may give some local elasticity. This structure can be compared with *Apis mellifera* hives (Sandeman et al. 1996) where pieces of comb even bounded by frames on all sides still carry a low-frequency signal of about 15 Hz, while higher-frequency displacements of >60 Hz could not be detected four cells away from the source (Sandeman et al. 1996). Low-frequency signals in Western honeybees detected from the comb site are known for producing a grooming invitation dance, when workers stand stationary vibrating their bodies from side to side at a frequency of 4 Hz for 10 s (Land and Seeley 2003). It is also known that vibrations are transmitted in the plane of the comb face (Michelsen et al. 1986) when produced by ‘beggar’ bees which press their thoraces down onto the comb while producing a 320-Hz vibration by the thoracic musculature. Here, displacements of up to 1.5 μm at right angles to the plane of the comb face can be measured even a few centimetres, but not more, away from those bees. Such traits in *A. mellifera* combs accord with the physical properties of an elastic mechanical plate which acts as a highly damped oscillator.

In contrast to *A. mellifera*, giant honeybees produce the oscillation of the central comb by the upstrokes of the abdomens of shimmering bees which happens synchronized and cascaded predominantly at the nest surface. These mechanical waves propagate across the nest but are damped down by the multiple layers of the bee curtain before they reach the comb. The elastic mechanical plate hypothesis predicts here that the comb would oscillate at its local natural frequency bands, which may vary regarding the consistence of comb cells (Sandeman et al. 1996). Furthermore, the locally provoked vibrations should show displacements at different positions of essentially similar magnitude.

This aspect of an elastic mechanical plate is supported by the spectral properties of the comb of the experimental nest, in particular because of the occurrence of two main frequency bands, one slightly above 2 Hz and the other at 6 Hz (Fig. 6a–e). These two bands happened irrespective whether the nest was investigated under *quiescent* or *arousal* conditions (provoked by shimmering waves or by mass flight activity) and whether the vibrations had been detected near the attachment zone (position S1; Fig. 1) or near the lower rim zone (position S3). It also seems as if mass flight activity provoked vibrations at the higher frequency band rather than at the lower one, whereas shimmering waves mainly drove the comb at the lower frequency band. Although the data displayed in Fig. 5 were ephemeral, they document the stable character of both frequency bands, in particular at small vibration amplitudes.

### Magnitude of mechanoreceptive signalling

The behavioural traits of the mechanical oscillations at the comb generated by shimmering raise two further questions: First, do the vibrations gain sufficient power to achieve signal value; in other words, are they sufficient to stimulate the curtain bees on the side contra-lateral to the threatened one? If the shimmering process has evolved (in addition to its anti-predatory goals, cf. Kastberger et al. 2008) to enforce colony-intrinsic communication, it should accelerate the comb by the wave-like flashes at intensities which exceed the sensory threshold of honeybees. In *A*. *mellifera*, the most sensitive frequencies were found (Sandeman et al. 1996) between 30 and 100 Hz with threshold displacements (*D*_th_) of 2 μm and a threshold sensitivity of *D*_th_ = 6.2 μm at 10 Hz. Extrapolation of the empirical data (Sandeman et al. 1996) to lower frequencies results in *D*_th_ = 8.97 μm for a 2-Hz vibration which corresponds to a threshold acceleration of 0.0355 mm/s^2^.

In *A. dorsata* nests, the vibration spectrum of the central comb, based on a cycle-related analysis during shimmering waves, revealed supra-threshold displacements of more than 9 μm per natural cycle of comb vibrations in 51.08 % of the observation time (Fig. 7), but still 10 % of vibration cycles had the fivefold value of 45 μm and, therefore, accelerate the comb by more than 0.2 mm/s. These vibrations were only present under ongoing shimmering activity but not after its termination and differed significantly in their dislocation spectra between the recording positions (*centre*, *att*) in the *arousal* phase of the experiments.

This means that shimmering flashes do have the potential to provoke comb vibrations as supra-threshold cues for the curtain bees. Due to the pendulum effect, we can expect that factually all curtain bees are able to sense this mechanical signal irrespective of their engagement in shimmering, practically in all layers of the curtain, and on both sides of the comb, but with two restrictions: first, these colony members who are positioned at the lower portions of the bee curtain would perceive the comb vibrations stronger than those positioned near the attachment zone; and second, the loose coupling of the bee curtain with the comb expectedly damps the impact of comb oscillations, in particular for the outer layers of curtain bees. Therefore, the cohorts near the comb could sense such vibrations more strongly than colony members at the outer layers of the bee curtain.

Summarizing, the findings not only support the pendulum’s hypothesis for the impact of the pulsed forces of shimmering on the comb of giant honeybee nests but also show that the elastic mechanical plate hypothesis matches the conditions under more stochastic forces such as under the conditions of mass flight activity (Kastberger et al. 1996).

## Conclusions

A giant honeybee (*A. dorsata*) nest comprises, from a physics viewpoint, a central comb as a stiff plate, attached to a solid substrate and covered by the multilayered bee curtain. Shimmering generates repetitive wave-like flashes in the mechanical domain and provokes vibrations which operate the comb as a driven under-damped oscillator. The repetition rate of shimmering waves (*F*_sh_ = 0.957 Hz) is ≈1 Hz lower than the basic frequency of the comb (*f*_c_ = 2.112 Hz), which determines the powering of the comb as a driven oscillator. The oscillation properties of the comb match the model of a mechanical pendulum under the pulses of shimmering waves, whereas under the much weaker and diffuse forces of mass flight activity or even under general quiescence of the nest, the comb shows with two frequency bands, a virtually harmonic property indicative for the performance as an elastic plate. Shimmering waves deliver forces upon the comb above the sensory threshold of honeybees, sufficiently strong for being perceived by worker bees throughout the bee curtain. The pendulum principle may provide mechanical information to those members of the bee curtain who are assembled nearer to the lower nest in the rim zone, while the elastic plate properties mediate shimmering-provoked vibration to those members who are in direct contact with the comb. The findings support the colony-intrinsic communication hypothesis that shimmering serves as a fast way to inform the members of the bee curtain about the momentary defensive state of the nest.

## Electronic supplementary material

Below is the link to the electronic supplementary material.

Online Resource 1 (PDF 258 kb)

Online Resource 2 Movie 1 *. Motion at the nest surface and comb vibrations under in the arousal (pink background) and quiescence (blue background) phase of the experiments*. First data panel: the horizontal position of the dummy wasp (HOR dw) over time during three passes across and back in the front of the nest (which defined the arousal phase in the experiment) from position 0 m at the left side to position 1 m at the right side as depicted in the images of Figs. 1 and 3. Second data panel (Motion [px]): the time course of motion activity due to shimmering (*A*_sm_) at the surface of the experimental nest scaled in px area. The peak performance was caused by the movement of the dummy wasp (pictured above left). Third data panel (Vibration [mm]): the oscillation scaled as dislocation (*D*_c_) over time of the central comb in mm, as assessed by LDV with the reflective end of the rod as target at the centre position of the nest (see inserted spot at the end of the rod in the image above left). The video film above right simultaneously displays the difference images of the film as shown in the film panel at the left. The sum of white spots quantifies the *A*_sm_ value of the second panel. The position of the red running cursor is synchronized with the images of both the films above (cf. Fig. 4) (AVI 9865 kb)
