# Supplementary material for: Social waves in giant honeybees (Apis dorsata) elicit nest vibrations
Source: Naturwissenschaften. 2013 May 31;100(7):595–609. doi: 10.1007/s00114-013-1056-z (PMC3696463; doi:10.1007/s00114-013-1056-z)
Supplement: Supplementary file 1 — (PDF 258 kb) [file 114_2013_1056_MOESM1_ESM.pdf]

Online Resource 1

Additional comments for the paper "Social waves in giant honeybees (Apis dorsata) elicit nest vibrations"

by Gerald Kastberger, Frank Weihmann, Thomas Hoetzl

Assuming a physical pendulum function for a giant honeybee nest

The model view of a physical pendulum can be adopted for a typical giant honeybee (*Apis dorsata*) nest out of three reasons: (a) in Chitwan (Nepal), nests are typically suspending from house balconies or from a limb of a tree. They are attached to this solid structure only with their upper part. (b) From a physical viewpoint, a nest is an elastic compound body, shaped by a stiff comb (because its cells are filled up with watery content, such as honey or larvae) with a cover of bees representing the bee curtain. It swings around a pivotal axis at the attachment line if provoked by gusts of wind, or by mechanical shocks in the structure to which it is attached. Lastly (c), the inertial force of a shimmering wave buffets this virtual pendulum asymmetrically at the threatened nest side.

A physical pendulum such as the comb (c) of an *A. dorsata* nest is a weighted "rod" of given length swinging from a pivot (Nave 2001) whose period T_c, respectively its natural frequency ω_c, depends on its moment of inertia [I_c] around the pivot point, on its mass [M_c] and on the length of the functional rod [L_c], the distance between the pivotal point and the gravity point of the comb (***Equations S1***).

Equation S1a: $T_c = 2\pi\ (I / M_c\, g\, L_c)^{0.5}$

Equation S1b: $\omega_c = 1/T_c$

For small dislocation angles of the comb (such as $\Theta_c \approx \sin \Theta_c$), the rotational paradigm of a physical pendulum can be converted, for the purpose of simplification, into linear relations. Therefore, the shimmering-based dislocation force F_D (***Equation S2a***, and the work W_D (***Equation S2b***) can be calculated in the model by substituting the empirical values (M_c = 30-50 kg; L_c = 30 cm which is represented by the distance between the pivotal axis and the position centre; for the assessment of T_c, see Fig. 4B,D). Both force and work aspects consider the situation at the comb.

Equation S2a (force aspect): $F_D = M_c\, a_c \sin \Theta_c$, with $\Delta l_c = L_c \sin \Theta_c$ and $\Delta t_c = T_c/2$; $a_c = \Delta l_c / \Delta t_c^2$

Equation S2b (work aspect): $W_D = F_D\, L_c \sin \Theta_c$

Generally, the driven rotational movement of a comb regards the relationship between the net external torque τ_c and the angular acceleration α_c (***Equation S3a***) which is of the same form as Newton's second law whereas the

net external torque can be substituted by ***Equation S3b***. This torque value considers the force at the surface layer of the nest but not at the comb.

Equation S3a: $\tau_c = I\ \alpha_c$ (with [kg m^2 / s^2] = [Joule])

Equation S3b: $\tau_c = r_c\ F_{sh} \sin \Theta_c$ [Joule] with F_{sh} as the force applied by shimmering; r_c, the radius from the axis of rotation to the point of application of the force, here L_c; Θ_c, the dislocation angle

Assessment of dislocations and accelerations of comb vibrations (frame-based dislocation spectrum)

In this approach we used the empirical data (n_{exp} = 21; n_{ff} = 132 321 time intervals of 0.02 s) to calculate the comb dislocations (D_c) and accelerations (a_c) for both measurement positions (*center*, *att*) in the arousal and quiescence phases of the experiments. The sample values (a_c, D_c) were based on the inter-frame intervals Δt (***Equation S2***) and sorted into 32 categories (***Fig. S1B***). The distributions of the mean rates of inter-frame intervals (${}_{rel}n_{ff}$, ***Equation S4***) were fitted by exponential regression ($y = c.e^{d.x}$, with $x = D_c$ [µm/0.02 s] respectively x = ac [mm/s^2], and y = ${}_{rel}n_{ff}$).

Equation S4: ${}_{rel}n_{ff} = n_{ff}$ [step] / N_{ff} with *step* as category of the abscissa in ***Fig. S1A***, and N_{ff} as the number of all inter-frame intervals per measurement position (*center*, *att*)

This approach considers the physical properties of the comb as assessed by the LDV (***Fig. S1B***) under a digital low-pass filter regarding the interframe length of Δt =20 ms at 50 Hz. It shows that 63.06 per cent of all cases happened at lower dislocations (D_c<1.50 µm per 0.02s) respectively lower accelerations. The regression function for the *centre* position yielded higher occurrences of dislocation values for D_c>1.50 µm per 0.02s than at position *att* ($P < 0.001$; Mann-Whitney Test; ***Fig.S1B***; n_{exp_centre}= 9; n_{ff_centre} =56 619; n_{exp_att}=11, n_{ff_att} =88 074; n_{cat}=27). Consequentially, the median dislocations were greater at position *centre* than at position att (D_{c_centre} =2.624; D_{c_att} =2.189 µm per 0.02s).

The data have been assessed in inter-frame steps of 20 ms (***Fig. S1***) and, therefore, mimic higher acceleration values than those which obviously occur physically at the comb. They include *arousal* and *quiescent* conditions and, importantly, demonstrate that the vibration records can be statistically discerned between the positions *center* and *att* for dislocations D_c<1.50 µm per 0.02s. Although only of theoretical relevance, these facts can be particularly used for the comparison with mathematical noise models (see below). It can be demonstrated by those models using the basic ("natural") oscillation frequency of the experimental comb (f_c = 2.112 Hz) that the slopes of dislocation and acceleration spectra (***Fig. S1B***) is not only a function of the strength of oscillation, but also a function of noise. ***Fig. S2*** exemplifies this for two forms of noise (***Equations S5***: k_{noise}*m_{rnd}) by calculating the occurrence of dislocation values in discrete time intervals (Δt) of the sinusoidal function H = A * sin (t_0+Δt_{ff} +1/f_c)+B whereby H_a and H_b (***Equations S5***) differ only in the randomized amplitudes (A,B).

Equation S5a: H_a : A= k_{noise}*m_{rnd}, t_0 = 0; B = 0

Equation S5b: H_b :, A = 1; t_0 = 0; B= k_{noise}*m_{rnd}

with Δt_{ff} = 0.02s, fps = 50 Hz frame rate; f_c = 2.112 Hz as the natural frequency of the comb; $t_{0,}$ the start time of the observation; m_{rnd}, random values < 1.0, k_{noise}, factor of randomness

The data of both noise models were sorted in the same way into acceleration categories as done for the empirical data under ***Fig. S1B***. Under normalized scales, the slope-relevant parameter d of the regression $y = c\,e^{dx}$ (***Figs. S1A***, ***S2B₁***) depends on the factor k_{noise} in a characteristic way. The function H_a envelopes noise (k_{noise}*m_{rnd}) within the sinus oscillation, and the function H_b adds up noise above the sinus curve. In both functions noise decreases the probability of occurrence of higher magnitudes of dislocations. If this situation had affected the results in ***Fig. S1A*** (besides the dependency on vibration amplitude) higher level of noise at position *att* could have been the reason for the lower dislocation spectrum.

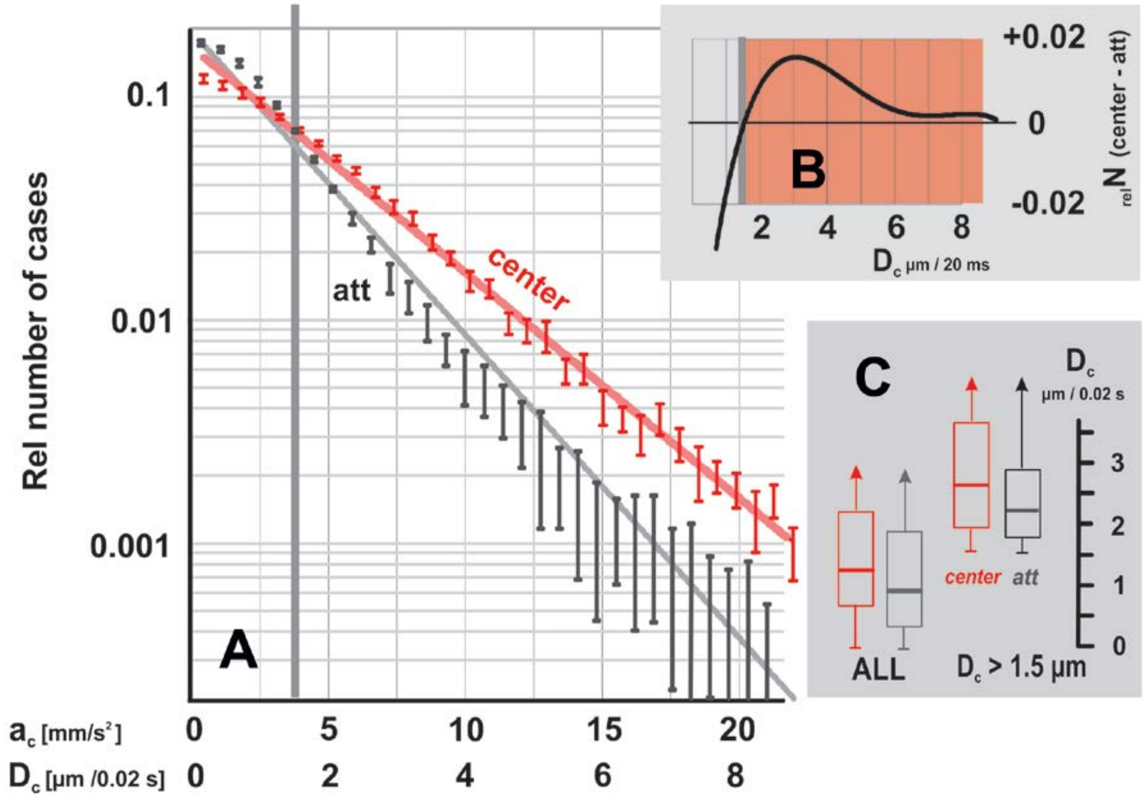

Fig. S1 Frame-based dislocation spectrum at the comb in the experimental Apis dorsata nest as provoked by shimmering waves. For measurement position *centre*, samples are shown of motion activity at the nest surface during shimmering (panel A_1) and the corresponding vibrations at the comb (panel A_2). (A) Spectra of accelerations respectively dislocations of the comb of the experimental *Apis dorsata* nest as provoked by shimmering waves; ordinate, relative numbers ($_{rel}$N) of interframe intervals (0.02 s) in which the vibration corresponded with the respective class of acceleration (a_c) respectively dislocation (D_c). The two recording positions (*centre*: pink symbols, N_{centre} = 88 074; *att*: grey symbols, N_{att}=56 619 time intervals) showed

significant differences (P<0.01, Student test) for D_c<1.50 µm per 0.02s; regression functions $y = c.e^{d.x}$ with c_{centre}=0.1623, d_{centre}=-0.161; R^2=0.9942; c_{att}=0.1911, d_{att} = -0.220; R^2=0.9883) in relation to the higher rates of acceleration a > 5 mm/s^2; vertical bars indicate ±SEMs, their mid points indicate the arithmetical means of relative number of cases $_{rel}N$; n_s = 21 experimental sessions. (B) Plot of the difference $\Delta_{rel}N$ between the two recording positions ($\Delta_{rel}N$ = $_{rel}N_{centre}$ - $_{rel}N_{att}$) corresponding to the 32 acceleration (dislocation) steps (see panel B). (C) Box and Whisker dislocation plots at both measurement positions (*centre*, *att*); ALL refers to the entire dislocation spectrum; D_c > 1.5 µm, refers only to those intervals with dislocations of D_c > 1.5 µm per s; arrows symbolise that the maximum recorded dislocation value was D_c = 8.75 µm per s (see panel B)

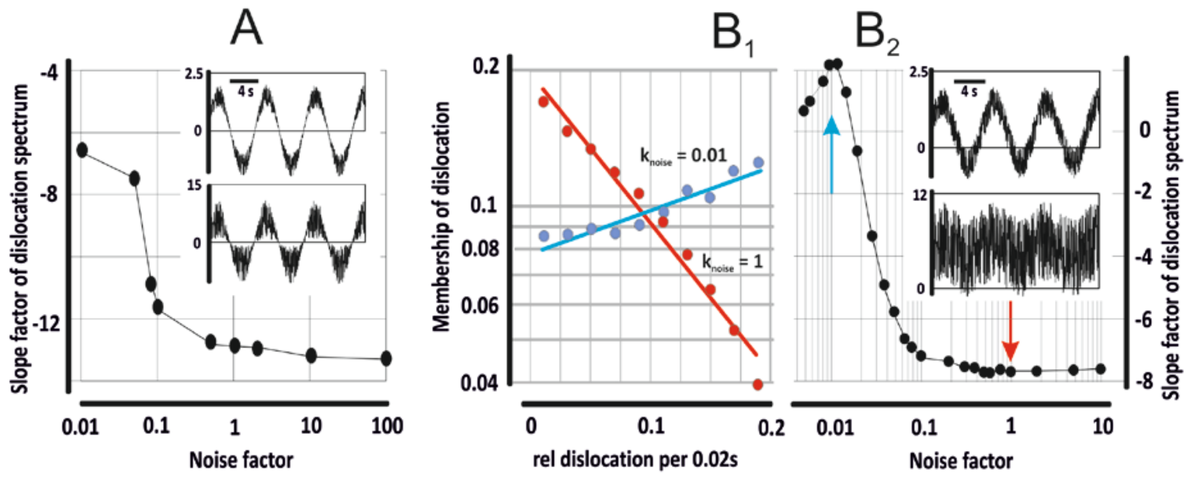

Fig. S2 How noise may influence the shape of the dislocation (acceleration) spectrum of an oscillator. Two sinusoidal functions H_a and H_b (Equations 8a,b) produce two paradigms of noise. The function H_a (see inset A) envelopes noise within the sinus curve and the function H_b (see inset B) adds up the noise above the sinus curve. (A) Slope factor of dislocation spectrum decreases with increasing noise. This is exemplified for H_b for two correlations; panel B_1 demonstrates the spectrum of rel dislocations per frame interval (0.02 s) for two noise factors (k_{noise} = 0.01 and 1.0); abscissa, classes of relative magnitude of dislocation values; ordinate, the membership to a dislocation class. Both models show that for higher noise levels (e.g. k_{noise} > 0.1; see panel B_2) the probability of the occurrence of larger dislocations is lower (consider for this the relative scale of dislocation). For further details, see text
